# Supplementary material for: A universal sequencing read interpreter
Source: Sci Adv. 2023 Jan 4;9(1):eadd2793. doi: 10.1126/sciadv.add2793 (PMC9812397; doi:10.1126/sciadv.add2793)
Supplement: Supplementary file 1 — Figs. S1 to S4 [file sciadv.add2793_sm.pdf]

Science Advances
AAAS

Supplementary Materials for

A universal sequencing read interpreter

Yusuke Kijima *et al.*

Corresponding author: Nozomu Yachie, nozomu.yachie@ubc.ca

Sci. Adv. **9**, eadd2793 (2022)
DOI: 10.1126/sciadv.add2793

The PDF file includes:

Figs. S1 to S4
Legends for tables S1 to S4

Other Supplementary Material for this manuscript includes the following:

Tables S1 to S4

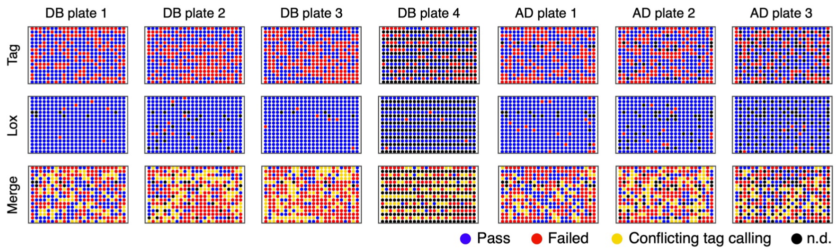

Fig. S1. Identification of high-quality clonal BFG-Y2H barcode cassette samples.

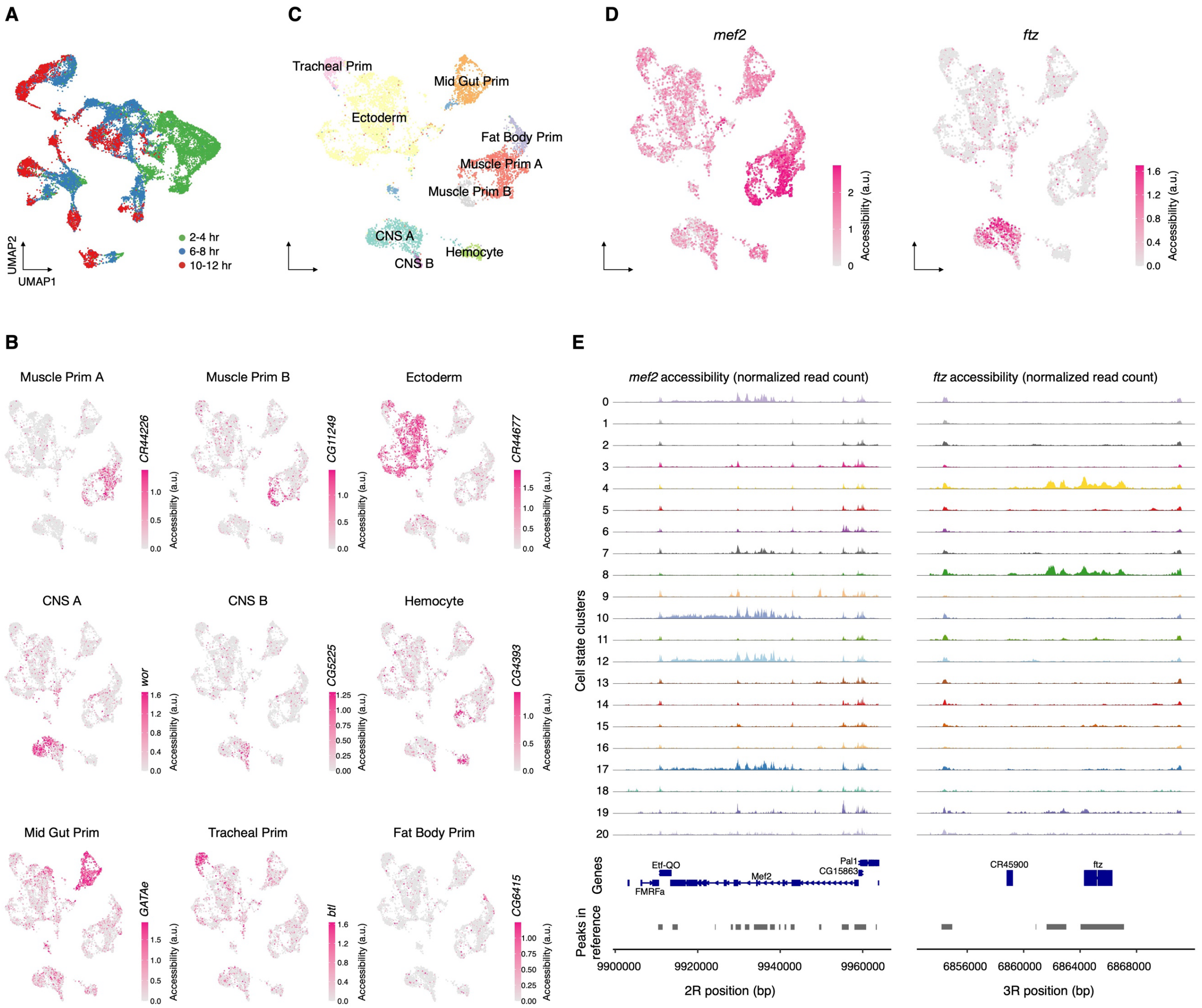

Fig. S2. Analysis of sci-ATAC-seq reads translated for 10X Cell Ranger ATAC. (A) Two-dimensional UMAP embedding of the sci-ATAC-seq datasets processed by its original pipeline for Drosophila embryo 2 to 4, 6 to 8, and 10 to 12 hours after egg laying. **(B-E)** Analyses of the same dataset translated by INTERSTELLAR and analyzed by 10X Cell Ranger ATAC. **(B)** Single-cell chromatin accessibilities of marker genes. **(C)** Cell state annotations. **(D)** Single-cell chromatin accessibilities of *mef2* and *ftz*. **(E)** Chromatin accessibilities of *mef2* and *ftz*-encoding regions in different cell state clusters represented in Fig. 3B.

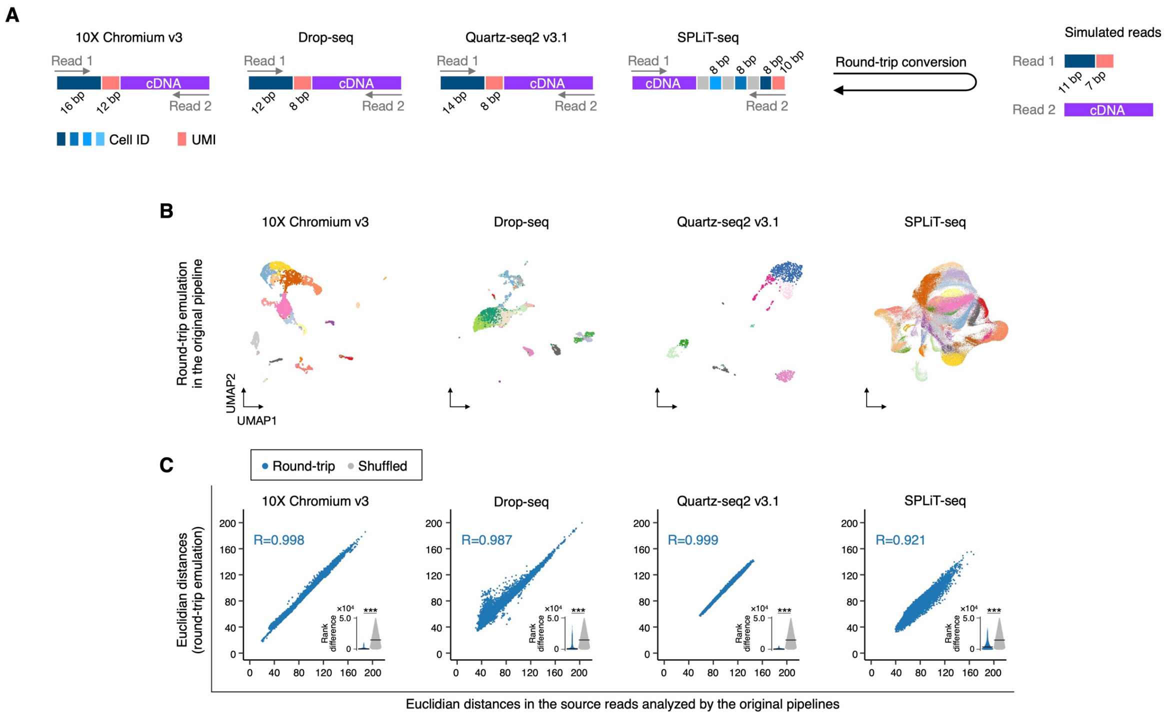

Fig. S3. **Round-trip conversion of scRNA-seq reads through a synthetic read structure with smaller information capacity. (A)** Each scRNA-seq read structure was converted into a simulated read structure with 11-bp cell ID and 7-bp UMI using INTERSTELLAR. The simulated reads were then translated back into the original read structure. **(B)** Two-dimensional UMAP embeddings of the round-tripped reads analyzed by their proprietary software tools. Cell cluster annotations were adapted from the original read analysis shown in Fig. 5B. **(C)** Correlation in Euclidean distance of two cells in high-dimensional transcriptome space between the original datasets and those produced by the round-trip conversion. For each dataset, Euclidean distances in the gene expression count matrix were measured for 50,000 randomly sampled cell pairs. The inset sina plots represent rank difference distribution in the Euclidean distance of the same cell pairs before and after translation. The crossbar represents the median.

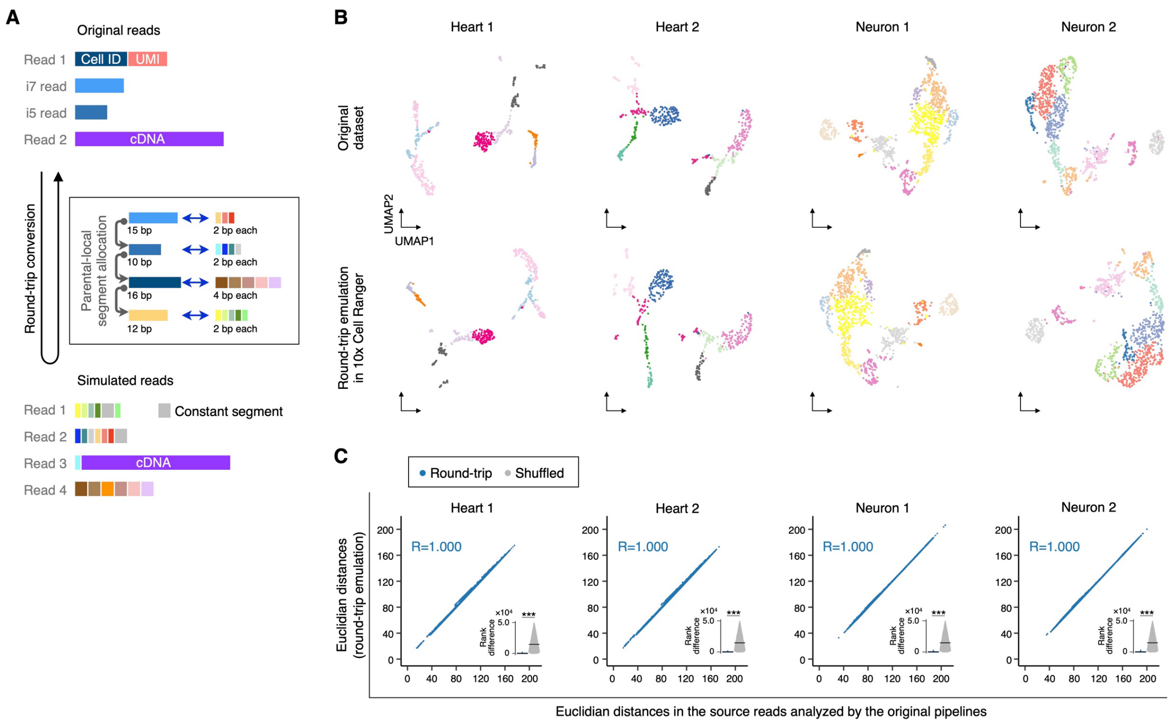

Fig. S4. **Round-trip conversion of scRNA-seq reads through a highly complex read structure. (A)** Four 10X Chromium V3 scRNA-seq libraries each with a unique library index pair were combined and transformed into a simulated pooled library of a complex read structure using INTERSTELLAR. The simulated library was then translated back into a pooled library of the Chromium V3 read structure. **(B)** Two-dimensional UMAP embeddings of four original scRNA-seq datasets (Heart 1K Lane 1, Heart 1K Lane 2, Neuron 1K Lane 1, and Neuron 1K Lane 2) and those by the round-trip conversion. Cell cluster annotations of single cells obtained by the original pipelines were applied to the round-trip conversion results. **(C)** Correlation in Euclidean distance of two cells in high-dimensional transcriptome space between the original datasets and those produced by the round-trip conversion. For each dataset, Euclidean distances in the gene expression count matrix were measured for 50,000 randomly sampled cell pairs. The inset sina plots represent rank difference distribution in the Euclidean distance of the same cell pairs before and after translation. The crossbar represents the median.

List of Supplementary Tables

**Table S1**	Distribution of Slide-seq positional barcode numbers assigned per Visium position.
**Table S2**	Datasets used in this study.
**Table S3**	Primers used for RCP-PCR.
**Table S4**	Runtime measures for scRNA-seq read translations.
